# Supplementary material for: Reverse oxygen spillover triggered by CO adsorption on Sn-doped Pt/TiO2 for low-temperature CO oxidation
Source: Nat Commun. 2023 Jun 13;14:3477. doi: 10.1038/s41467-023-39226-6 (PMC10264398; doi:10.1038/s41467-023-39226-6)
Supplement: Supplementary file 1 — Supplementary information [file 41467_2023_39226_MOESM1_ESM.pdf]

Supplementary Information

Reverse Oxygen Spillover Triggered by CO Adsorption on Sn-doped Pt/TiO_2 for Low-Temperature CO Oxidation

Jianjun Chen[1#], Shangchao Xiong[2#,*], Haiyan Liu[1], Jianqiang Shi[1], Jinxing Mi[1], Hao Liu[1],

Zhengjun Gong[2], Laetitia Oliviero[3], Françoise Maugé[3], Junhua Li[1*]

[1]*State Key Joint Laboratory of Environment Simulation and Pollution Control, School of Environment, Tsinghua University, Beijing 100084, PR China*

[2]*Faculty of Geosciences and Environmental Engineering, Southwest Jiaotong University, Chengdu 610031, PR China*

[3]*Laboratoire Catalyse et Spectrochimie, ENSICAEN, Université de Caen, CNRS, 6 bd du Maréchal Juin, 14050 Caen, France.*

[#]**Author contributions**

These authors contributed equally.

***Corresponding author.**

Phone: +86 010 62771093

Email address: lijunhua@tsinghua.edu.cn (Junhua Li)
xiongshangchao@swjtu.edu.cn (Shangchao Xiong)

Content including: 4 Notes, 5 Tables and 23 Figures

Supplementary Note 1. Absence of heat and mass transfer limitations.

The absence of heat and mass transfer limitations were estimated by Mears criterion.

First, we ensured the plug-flow behavior by the following relations:

$$\frac{d_t}{d_p}=\frac{6\times10^{-3}m}{337.5\times10^{-6}m}\approx 18>10 \quad \text{(S1)}$$

$$\frac{L_b}{d_p}=\frac{2\times10^{-2}m}{337.5\times10^{-6}m}\approx 59>50 \quad \text{(S2)}$$

where d_t– reactor tube diameter, d_p – particle diameter, L_b – bed length.

Mears criterion for the interphase heat transfer limitations:

$$\frac{|\Delta H_R| r_{obs} R_p E_a}{R_g h T_b^2}<0.15 \quad \text{(S3)}$$

where ΔH_R – reaction heat (J mol^{-1}), r_{obs} – rate per catalyst volume (mol m_{cat}^{-3} s^{-1}), R_p – particle radius (d_p/2), E_a – activation energy (J mol^{-1}), R_g – ideal gas constant (J mol^{-1} K^{-1}), h – gas-solid heat transfer coefficient (W m^2 K^{-1}), T_b – temperature of bulk fluid (K).

$$h=\frac{2\lambda_g}{d_p} \quad \text{(S4)}$$

where λ – thermal conductivity of the gas,

$$\lambda_g = y_{CO}\lambda_{CO}+y_{O_2}\lambda_{O_2}+y_{N_2}\lambda_{N_2}=0.01(0.030)+0.01(0.032)+0.98(0.030)=0.03\frac{W}{m\,K} \quad \text{(S5)}$$

$$giving\; h=\frac{2\times0.03}{337.5\times10^{-6}}=178\frac{W}{m^2\,K} \quad \text{(S6)}$$

ΔH_R = 283×10^3 J mol^{-1}; r_{obs} = 10.26 mol m_{cat}^{-3} s^{-1}; R_p = 0.5×337.5×10^{-6} m; E_a = 29.8×10^3 J mol^{-1}; R_g = 8.314 J mol^{-1} K^{-1}; T_b = 373 K; The apparent (poured) density of the catalyst (sieved fraction): ρ_c = 1.32×10^6 g m^{-3}.

Mears criterion at 100 °C:

$$\frac{|\Delta H_R| r_{obs} R_p E_a}{R_g\, h\, T_b^2}=\frac{283\times10^3\times10.26\times\frac{337.5\times10^{-6}}{2}\times29.8\times10^3}{8.314\times178\times373^2}\approx 0.07<0.15 \quad \text{(S7)}$$

Therefore, our experimental conditions satisfy the Mears criterion for the absence of heat transfer limitations.

Mears criterion for the mass transport limitations:

$$\frac{r_{obs} R_p}{k_c C_{Ab}} < 0.15 \quad \text{(S8)}$$

k_c – mass transfer coefficient of the reactant (m s^{-1}), C_{Ab} – concentration of the reactant in the bulk gas phase (mol m^{-3}). Since Re << 1,

$$k_c \sim \frac{2D_{CO-N_2}}{d_p} \quad \text{(S9)}$$

D_{CO-N2} – diffusion coefficient of CO in N_2 (m^2 s^{-1}).

$$k_c = \frac{2 \times 3.04 \times 10^{-5}}{337.5 \times 10^{-6}} \approx 0.18 \frac{m}{s} \quad \text{(S10)}$$

C_{Ab} = 0.33 mol m^{-3}.

$$\frac{r_{obs} R_p}{k_c C_{Ab}} = \frac{10.26 \times \frac{337.5 \times 10^{-6}}{2}}{0.18 \times 0.33} \approx 0.03 < 0.15 \quad \text{(S11)}$$

Therefore, our experimental conditions satisfy the Mears criterion for the absence of external mass transfer limitations.

Experimental diagnostic tests

To validate that the catalytic data was obtained in the kinetic regime, we performed additional diagnostic tests using our most active catalyst – $Pt/Sn_{0.2}Ti_{0.8}O_2$. We varied the amount of catalyst loaded in the reactor and the overall flow, keeping the ratio between the flow rate and catalyst weight constant. As shown in Supplementary Fig. S1, the reaction rate profiles are nearly identical, meaning that the reaction kinetics is not affected by the transport phenomena.

Supplementary Note 2. H_2 pretreatment temperature and the valence of Pt

The H_2 pretreatment process utilized a 5% H_2 concentration and a treatment time of 1 h. Subsequently, $Pt/Sn_{0.2}Ti_{0.8}O_2$ was subjected to various treatment temperatures. Supplementary Fig. S2a demonstrates that $Pt/Sn_{0.2}Ti_{0.8}O_2$ treated at 300 °C exhibited the highest CO oxidation performance. Therefore, the designed $Pt/Sn_xTi_{1-x}O_2$ and Pt/TiO_2 catalysts were evaluated for CO oxidation after pretreatment in 5% H_2 at 300 °C for 1 h.

Previous studies have suggested a correlation between the valence state of surface Pt and the nanoparticle size of the catalyst. In general, the smaller the nanoparticle size of Pt clusters, the higher the oxidation state. For instance, research by Somorjai[1] has demonstrated that in Pt_{20} clusters (~0.8 nm), Pt is primarily present as Pt^{2+}, while in Pt_{40} clusters (~1.5 nm), Pt is predominantly present as Pt^0. However, the valence state of Pt clusters can also vary depending on the chemical environment, even when the particle size is the same. Ozturk et al.[2] found that Pt $4f_{7/2}$ peaks were located at 74.6 eV (representing primarily Pt^{4+}) and 73.3 eV (representing primarily Pt^{2+}) for Pt_{40} nanoparticles deposited as a thick and a thin layer, respectively. Ye et al.[3] observed that the Pt $4f_{7/2}$ peak presented at 73.0 eV for Pt_{30} nanoparticles, suggesting that in this case, Pt is mainly present in the form of Pt^{2+}. Therefore, in this study, XPS spectroscopy was utilized to investigate the oxidation state of surface Pt under different H_2 reduction temperatures and before/after H_2 reduction at 300 °C. As shown in Supplementary Fig. S2b, the $Pt/Sn_{0.2}Ti_{0.8}O_2$ catalyst surface contained a small amount of Pt^{4+} and a large amount of Pt^{2+} before H_2 reduction. After H_2 reduction at 300 °C, Pt^{4+} on the surface of $Pt/Sn_{0.2}Ti_{0.8}O_2$ was reduced to Pt^{2+}, and no further reduction from Pt^{2+} to Pt^0 was observed even at an increased reduction temperature of 500 °C, indicating that Pt^{2+} can remain stable on the surface of $Pt/Sn_{0.2}Ti_{0.8}O_2$. Furthermore, after H_2 reduction at 300 °C, Pt species on the surface of $Pt/Sn_{0.2}Ti_{0.8}O_2$, Pt/TiO_2-R, and Pt/TiO_2-A catalysts were all present mainly in the form of Pt^{2+} (Supplementary Fig. S3).

Supplementary Note 3. The valence state of the carrier cations (Ti and Sn)

In situ NAP-XPS results (Fig. 3a) indicate that the oxidation of Pt^{2+} to Pt^{4+} occurred concurrently with CO oxidation, implying that there was first the transfer of O_{latt} species to Pt sites, and then the oxidation of CO to CO_2 by the O_{latt} species at the Pt sites. Therefore, the valence state of the carrier cations (Ti and Sn) should reduce when O_{latt} species transferred to Pt sites. However, due to the content difference of about 2 orders of magnitude between Pt and carrier elements (Ti, Sn and O), it is very hard to observe the change of chemical states over the *in situ* NAP-XPS spectra of Ti 2*p*, Sn 3*d* and O 1*s*. The further limitation was that a serious charging effect occurred during the *in situ* NAP-XPS studies, leading to significant deviation of characteristic peaks of Pt 4*f*, Ti 2*p*, Sn 3*d* and O 1*s*. Therefore, all the characteristic peaks were calibrated by their main peak before data analysis. This calibration method allows us to analyze the appearance of new characteristic peaks (such as the appearance of the peaks ascribable to Pt^{4+}), but cannot analyze the shift of peak position. Namely, it is more difficult to determine the change of chemical states of Ti, Sn and O. As a result of the above reasons, when O_{latt} species transferred to Pt sites, it is impossible to determine the decrease of valence state of Ti and Sn species by *in situ* NAP-XPS studies.

Supplementary Note 4. Configurations of $Pt/Sn_{0.2}Ti_{0.8}O_2$ and Pt/TiO_2-R.

The most stable configurations of $Pt/Sn_{0.2}Ti_{0.8}O_2$ and Pt/TiO_2-R used in the DFT simulation were obtained from an *ab initio* molecular dynamics (AIMD) simulation, in which 20% of Ti in rutile TiO_2 was replaced randomly by Sn to simulate the $Sn_{0.2}Ti_{0.8}O_2$ support. Since the SAC-STEM images and XPS results indicate that the Pt species on $Pt/Sn_{0.2}Ti_{0.8}O_2$ and Pt/TiO_2-R were in the form of small PtO clusters, Pt_4O_4 clusters were set on the surface of $Pt/Sn_{0.2}Ti_{0.8}O_2$ and Pt/TiO_2-R to undergo the simulation process. These configurations of $Pt/Sn_{0.2}Ti_{0.8}O_2$ and Pt/TiO_2-R first underwent a 10 ps of AIMD simulation at 700 K. As shown in Supplementary Fig. S20, these two configurations kept stable during the AIMD simulation. Then, the configurations at 2, 4, 6, 8 and 10 ps of AIMD simulation were set as initial structure and optimized by Vienna Ab-initio Simulation Package (VASP) to obtain the most stable configurations of $Pt/Sn_{0.2}Ti_{0.8}O_2$ and Pt/TiO_2-R. The total energy of these configurations after optimization were listed in Supplementary Table S5. The total energy of the configurations underwent different AMID simulation periods were almost identical, indicating that the Pt_4O_4 clusters kept stable during AIMD simulation. The complete configurations of $Pt/Sn_{0.2}Ti_{0.8}O_2$ and Pt/TiO_2-R chosen for the subsequently simulations were shown in Supplementary Fig. S21.

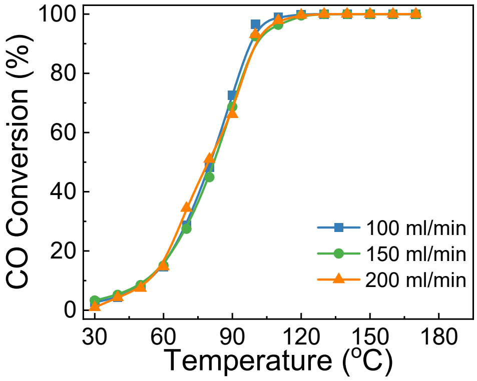

Supplementary Fig. S1 Diagnostic CO oxidation tests for Pt/$Sn_{0.2}Ti_{0.8}O_2$ sample with H_2 pretreatment. The reaction feed was 1%CO+1%O_2 in N_2 flow. Overall flow was varied from 100 to 200 ml/min. The weight of the catalyst loaded in the reactor was varied accordingly, keeping GHSV at 60 000 ml g_{cat}^{-1} h^{-1}.

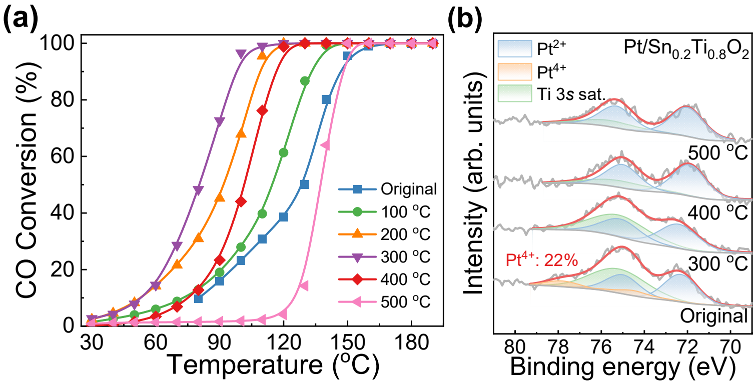

Supplementary Fig. S2 Screening of H_2 treatment temperatures. (a) Effect of different H_2 pretreatment temperatures on steady-state CO oxidation performance over Pt/$Sn_{0.2}Ti_{0.8}O_2$ with reaction feed of 1% CO, 1% O_2, N_2 balance and GHSV of 60 000 ml g_{cat}^{-1} h^{-1}. (b) Pt 4*f* XPS spectra of Pt/$Sn_{0.2}Ti_{0.8}O_2$ before and after pretreatment with H_2 at different temperatures.

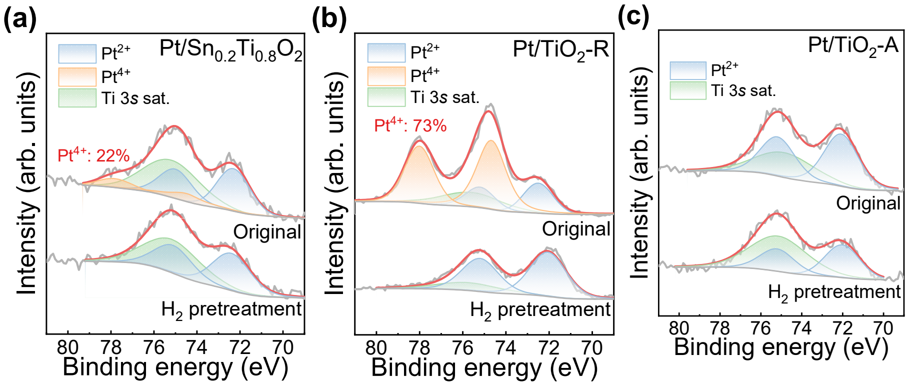

Supplementary Fig. S3 Pt 4*f* XPS spectra over original and 300 °C H_2-pretreated (a) Pt/$Sn_{0.2}Ti_{0.8}O_2$, (b) Pt/TiO_2-R and (c) Pt/TiO_2-A.

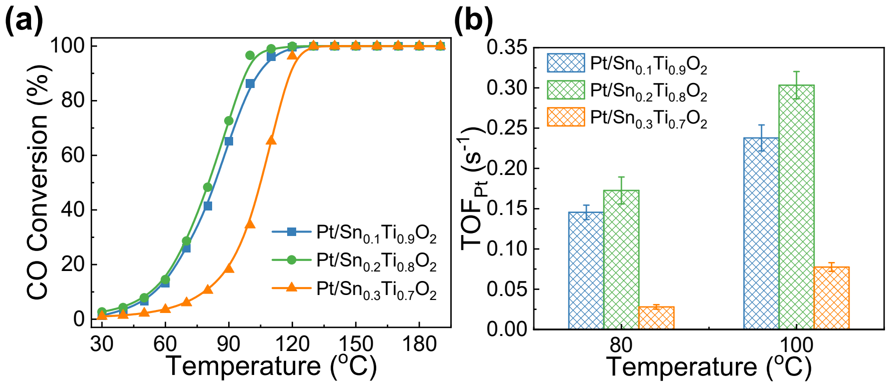

Supplementary Fig. S4 Steady-state CO oxidation performance over Pt/$Sn_xTi_{1-x}O_2$ with H_2 pretreatment. (a) CO conversion versus temperature plots with reaction feed of 1% CO, 1% O_2, N_2 balance and GHSV of 60 000 ml g_{cat}^{-1} h^{-1}. (b) TOF_{Pt} measured at 80 °C and 100 °C with CO conversion below 20%. The error bars represent standard deviations.

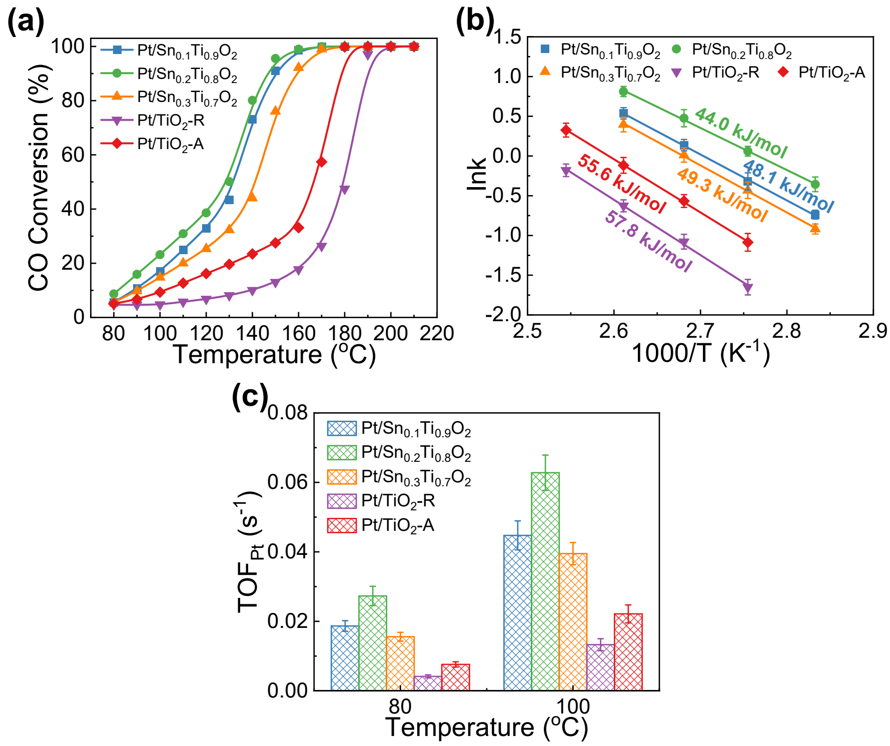

Supplementary Fig. S5 Steady-state CO oxidation performance over Pt/$Sn_xTi_{1-x}O_2$ and Pt/TiO_2 catalysts without H_2 pretreatment. (a) CO conversion versus temperature plots with reaction feed of 1% CO, 1% O_2, N_2 balance and GHSV of 60 000 ml g_{cat}^{-1} h^{-1}. (b) Arrhenius plots measured at 50–130 °C with CO conversion below 20%. (c) TOF_{Pt} measured at 80 °C and 100 °C with CO conversion below 20%. The error bars in (b) and (c) represent standard deviations.

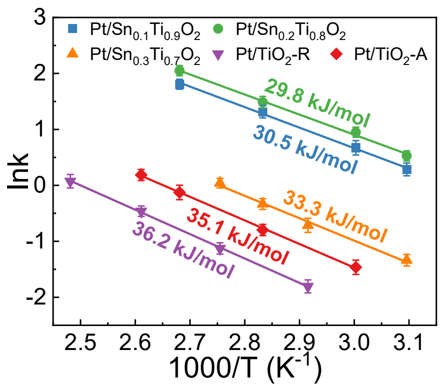

Supplementary Fig. S6 Arrhenius plots measured at 50–130 °C with CO conversion below 20% over Pt/$Sn_xTi_{1-x}O_2$ and Pt/TiO_2 catalysts with H_2 pretreatment. The error bars represent standard deviations.

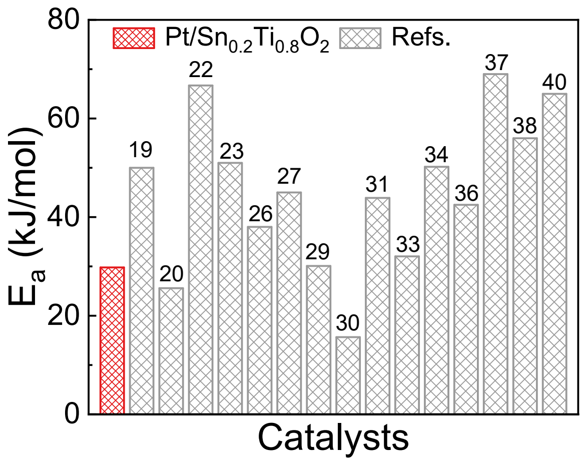

Supplementary Fig. S7 Comparison of E_a between Pt/$Sn_{0.2}Ti_{0.8}O_2$ and Pt-based catalysts (the numbers on top of the bars are the reference numbers of the referred works in main text).

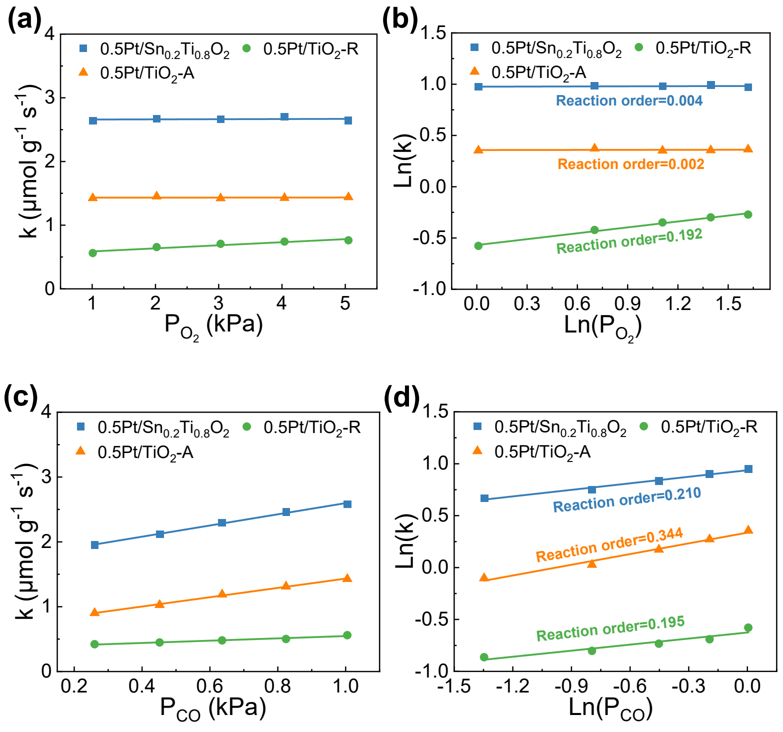

Supplementary Fig. S8 Reaction orders. CO oxidation rates as a function of (a) O_2 and (c) CO partial pressure; natural logarithm of CO oxidation rates as a function of natural logarithm of (b) O_2 and (d) CO partial pressure.

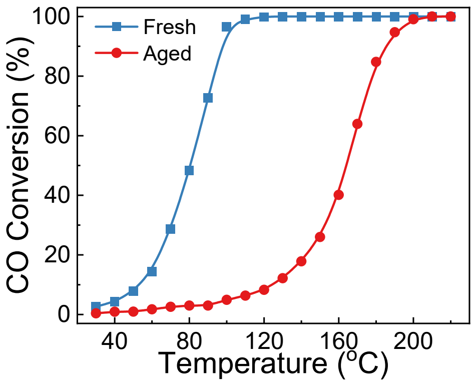

Supplementary Fig. S9 Hydrothermal aging effect on Pt/$Sn_{0.2}Ti_{0.8}O_2$. The reaction conditions of hydrothermal aging was 10% H_2O, N_2 balance, GHSV of 60 000 ml g_{cat}^{-1} h^{-1}, reaction temperature at 750 °C and reaction time of 9 h.

Supplementary Table S1 Comparison of CO oxidation performance between $Pt/Sn_{0.2}Ti_{0.8}O_2$ and the Pt-based catalysts in the references.

**Catalyst**	**Gas feed composition**	**Temperature (°C)**	**TOF (s^{-1})**	**E_a(kJ /mol)**	**Refs.[a]**	**Year**
$Pt/Sn_{0.2}Ti_{0.8}O_2$	1% CO, 1% O_2	80<br>100	0.172<br>0.303	29.8	This work	
1 wt% Pt NP/TiO_2	0.5% CO, 5% O_2	115	0.010		18	2022
1Pt2Bi-SiO_2	1% CO, 20% O_2			50	19	2021
1%wt Pt/CNT-600	1% CO, 20% O_2	100	0.0491	25.58	20	2021
0.5%wt Pt+LCT	2% CO, 10% O_2	180	0.486		21	2021
1%wt Pt_1/CeO_2_AT	1 % CO, 10% O_2			66.7	22	2021
0.5%wt Pt/CeO_2	1% CO, 4% O_2	80	0.20	51	23	2020
1%wt Pt NP/CeO_2-Al_2O_3 (Pt 600)	1% CO, 1% O_2	76	0.22		24	2020
0.25%wt Pt_1 SAC/CeO_x-TiO_2	1% CO, 4% O_2	200	0.093		25	2020
1%wt HO-Pt/TiO_2	1 % CO, 1 % O_2	70	0.11	38.0	26	2020
5% wt Pt-CC/Al_2O_3	1% CO, 1% O_2	120	0.028	45	27	2020
3%wt Pt_1 SAC/CeO_2	1 sccm of CO, 1.5 sccm of O_2, 75 sccm of He	180	0.072		28	2019
1%wt Pt NP/CeO_2	1.0 ml/min O_2, 1.5 ml/min CO, 75 ml/min He	80	0.101	30.1	29	2019
0.70%wt Pt_{NPs}/TiO_2−x	4% CO, 10% O_2			15.66	30	2019
0.42%wt Pt/$Ce_{0.8}Zr_{0.2}O_2$	1% CO, 21% O_2	100	0.156	43.9	31	2019
1.2%wt Pt-SA/A-Fe_2O_3	1% CO, 1% O_2	70	0.0687		32	2019
1wt %CeO_2-IMP-Pt	0.5% CO, 8%O_2	80	0.012	32.04	33	2018
1%wt Pt/TiO_2(B)	0.9% CO, 24% O_2	100	0.109	50.2	34	2018

0.2%wt Pt_1 SAC/m-Al_2O_3	2.5% CO, 2.5 % O_2	200	0.023		35	2017
1%wt Pt/CeO_2_S,	0.4% CO, 10% O_2			42.5	36	2017
0.025%wt Pt_1 SAC/TiO_2	1% CO, 1%O_2	200	0.117	69	37	2017
1% wt Pt/Al_2O_3+Polydedra Ceria	1.5 ml/min CO, 1 ml/min O_2, 75 ml/min He	225	0.15	56	38	2016
2%wt Pt/TiO_2-101	1000 ppm CO, 2.5% O_2	50	0.0075		39	2016
0.42%wt PtO_x/CeO_2 NWs-350	2% CO, 5% O_2	50	0.013	65	40	2015
0.3%wt Pt/TiO_2	1% CO, 1% O_2	40	0.0215		41	2013
0.18%wt Pt_1 SAC/θ-Al_2O_3	1% CO, 1% O_2	200	0.013		42	2013
0.17%wt Pt_1/FeO_x	1% CO, 1% O_2	27	0.136		43	2011

[a]For the references in this table please refer to the reference list of the main text.

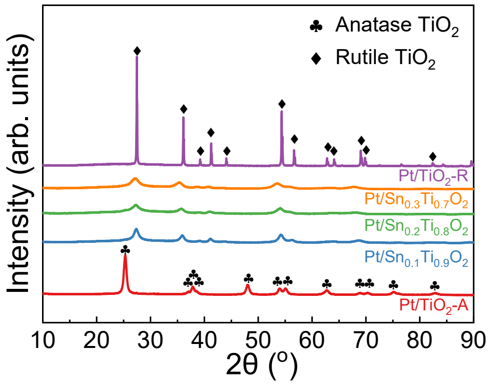

Supplementary Fig. S10 XRD patterns of Pt/$Sn_xTi_{1-x}O_2$ and Pt/TiO_2 catalysts.

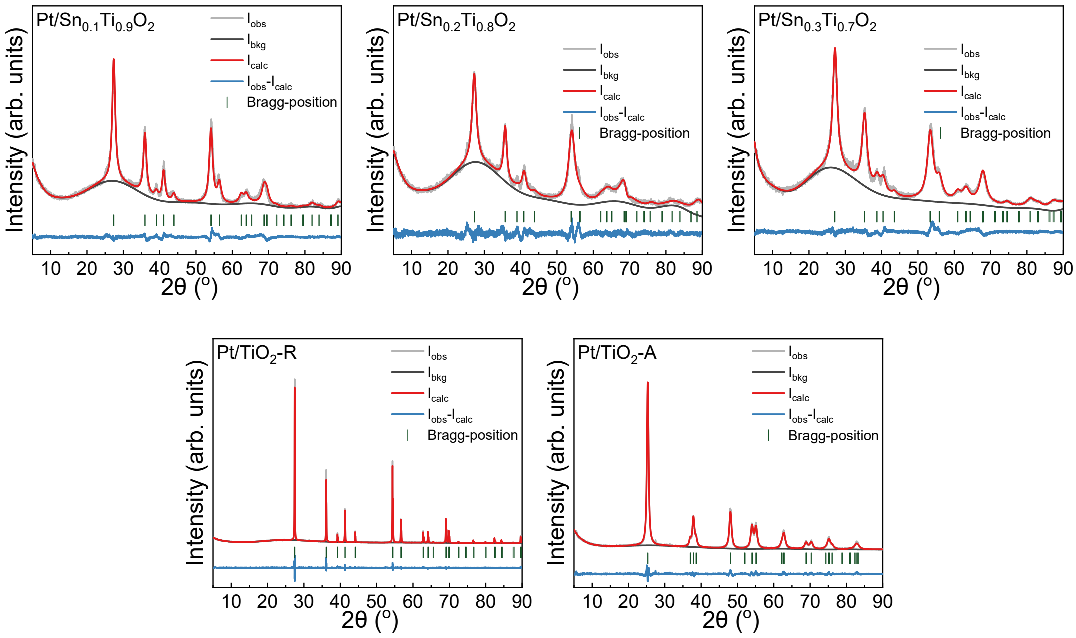

Supplementary Fig. S11 Rietveld refinement of XRD data for Pt/$Sn_xTi_{1-x}O_2$ and Pt/TiO_2 catalysts.

Supplementary Table S2 Refined parameters from Rietveld analysis of XRD data for Pt/$Sn_xTi_{1-x}O_2$ and Pt/TiO_2 catalysts.

Sample	Occupancy			Cell parameters	Rietveld Refinement Parameters		
	Ti	O	Sn		R_p/%	R_{wp}/%	χ^2
Pt/$Sn_{0.1}Ti_{0.9}O_2$	0.92	1	0.08	$a = b = 4.61$ Å<br>$c = 2.97$ Å<br>$\alpha = \beta = \gamma = 90°$	5.82	6.80	2.11
Pt/$Sn_{0.2}Ti_{0.8}O_2$	0.82	1	0.18	$a = b = 4.62$ Å<br>$c = 3.00$ Å<br>$\alpha = \beta = \gamma = 90°$	4.39	5.68	2.34
Pt/$Sn_{0.3}Ti_{0.7}O_2$	0.72	1	0.28	$a = b = 4.65$ Å<br>$c = 3.04$ Å<br>$\alpha = \beta = \gamma = 90°$	5.34	6.46	2.32
Pt/TiO_2-R	1	1	-	$a = b = 4.59$ Å<br>$c = 2.96$ Å<br>$\alpha = \beta = \gamma = 90°$	4.79	6.14	2.27
Pt/TiO_2-A	1	1	-	$a = b = 3.79$ Å<br>$c = 9.50$ Å<br>$\alpha = \beta = \gamma = 90°$	5.12	6.61	2.31

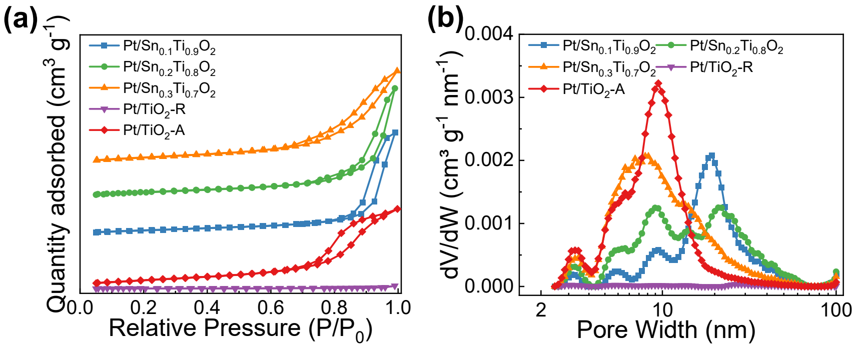

Supplementary Fig. S12 (a) N_2 adsorption and desorption curves and (b) pore size distributions of $Pt/Sn_xTi_{1-x}O_2$ and Pt/TiO_2 catalysts.

Supplementary Table S3 BET specific surface area (S_{BET}), total pore volume (V_p), and average pore diameter (D_P) of $Pt/Sn_xTi_{1-x}O_2$ and Pt/TiO_2 catalysts.

Sample	S_{BET}(m^2g^{-1})	V_p(cm^3g^{-1})	D_P(nm)
$Pt/Sn_{0.1}Ti_{0.9}O_2$	59.7	0.350	14.4
$Pt/Sn_{0.2}Ti_{0.8}O_2$	69.3	0.375	11.8
$Pt/Sn_{0.3}Ti_{0.7}O_2$	87.5	0.260	11.6
Pt/TiO_2-R	2.7	0.009	5.3
Pt/TiO_2-A	85.7	0.252	8.3

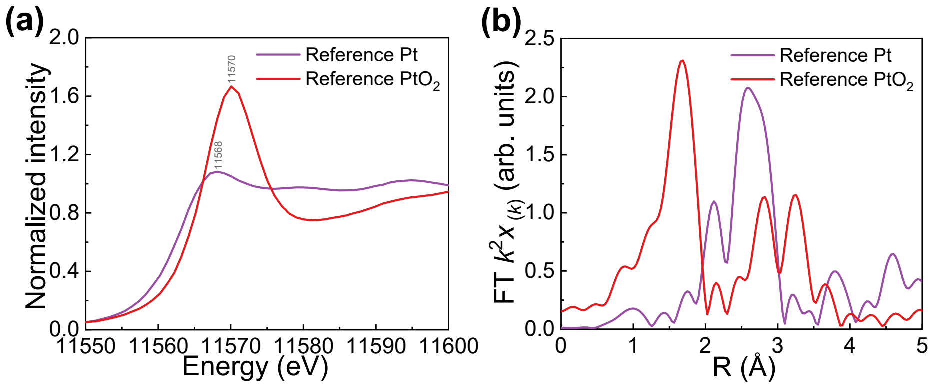

Supplementary Fig. S13 (a) XANES spectra of references Pt and PtO_2 over Pt L_3-edge. (b) The magnitude component of the k^2 weighted FT-EXAFS data of references Pt and PtO_2 over Pt L_3-edge.

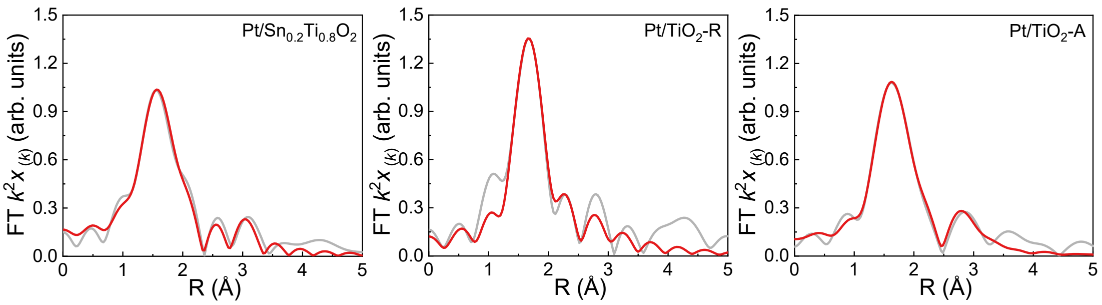

Supplementary Fig. S14 Raw and fit curves of the k^2 weighted FT-EXAFS data of Pt L_3-edge over Pt/$Sn_{0.2}Ti_{0.8}O_2$, Pt/TiO_2-R and Pt/TiO_2-A. Gray and red lines represent the raw and fit curves, respectively.

Supplementary Table S4 EXAFS fitting results of Pt L_3-edge over Pt/$Sn_{0.2}Ti_{0.8}O_2$, Pt/TiO_2-R and Pt/TiO_2-A. *CN*: coordination numbers; *R*: bond distance; σ^2: Debye-Waller factors; ΔE_0: the inner potential correction. *R* factor: goodness of fit. S_0^2 was set to 0.86, according to the experimental EXAFS fit of Pt foil reference by fixing *CN* as the known crystallographic value.

Sample	shell	*CN*	*R*(Å)	σ^2	ΔE_0	R factor
	Pt-O	4.5±0.3	1.99±0.03	0.0092		
Pt/$Sn_{0.2}Ti_{0.8}O_2$	Pt-Pt	1.4±0.5	2.70±0.03	0.0021	11.7±1.7	0.0159
	Pt-O-Sn	1.1±0.4	3.00±0.03	0.0019		
Pt/TiO_2-R	Pt-O	3.4±0.3	2.03±0.01	0.0017	17.8±1.6	0.0179
	Pt-Pt	1.4±0.5	2.79±0.02	0.0027		
	Pt-O	4.1±0.3	2.01±0.02	0.0065		
Pt/TiO_2-A	Pt-Pt	1.2±0.4	2.73±0.03	0.0019	14.8±1.8	0.0103
	Pt-O-Pt	3.4±1.4	3.56±0.05	0.0022		

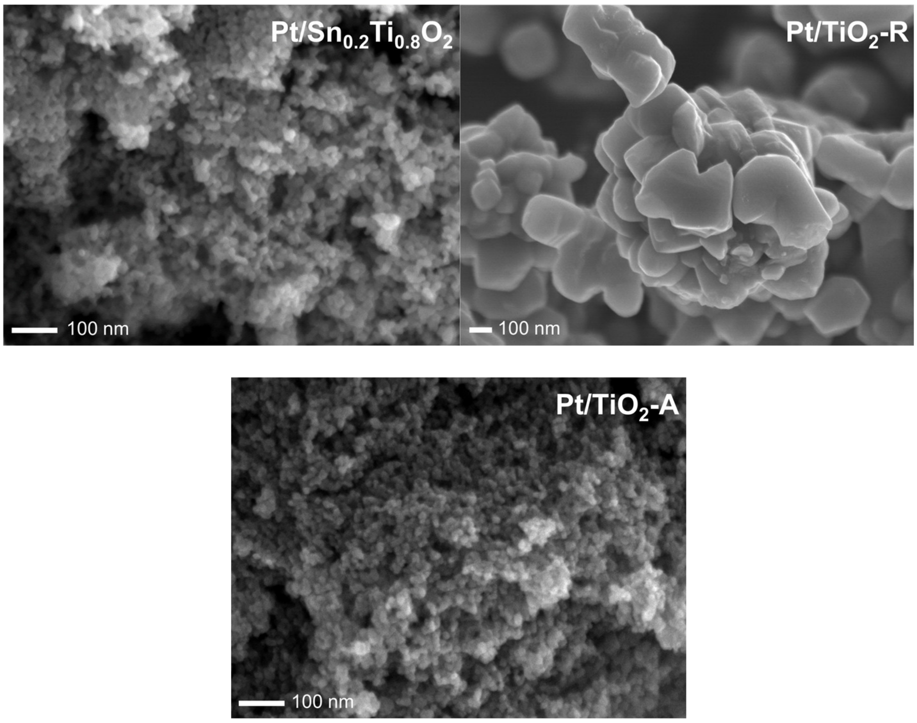

Supplementary Fig. S15 SEM images of Pt/$Sn_{0.2}Ti_{0.8}O_2$, Pt/TiO_2-R and Pt/TiO_2-A.

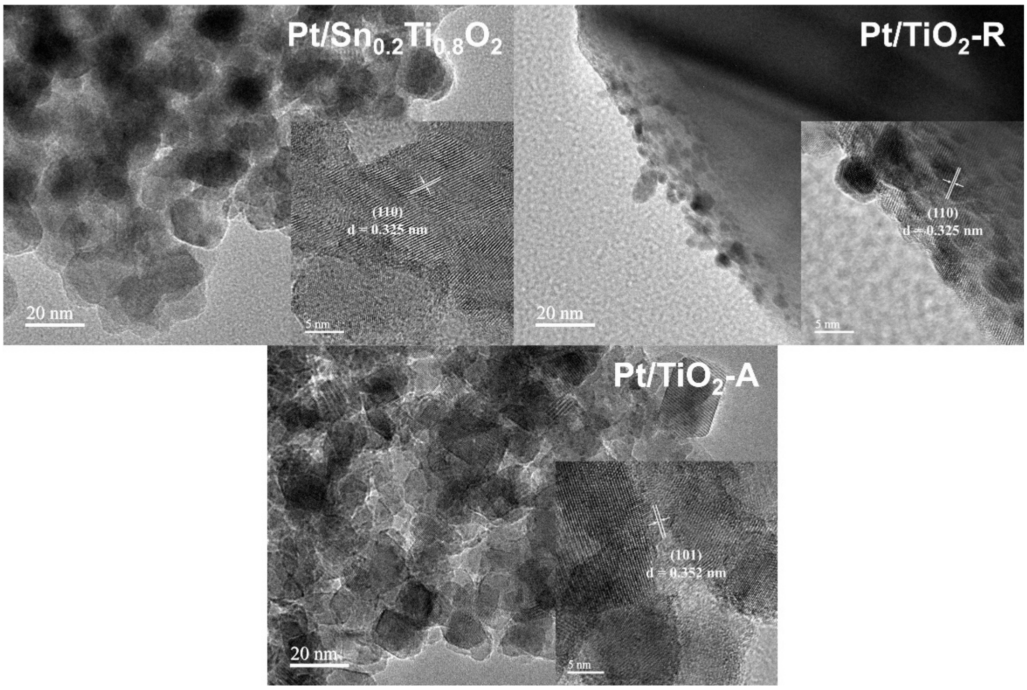

Supplementary Fig. S16 TEM and HR-TEM images of Pt/$Sn_{0.2}Ti_{0.8}O_2$, Pt/TiO_2-R and Pt/TiO_2-A.

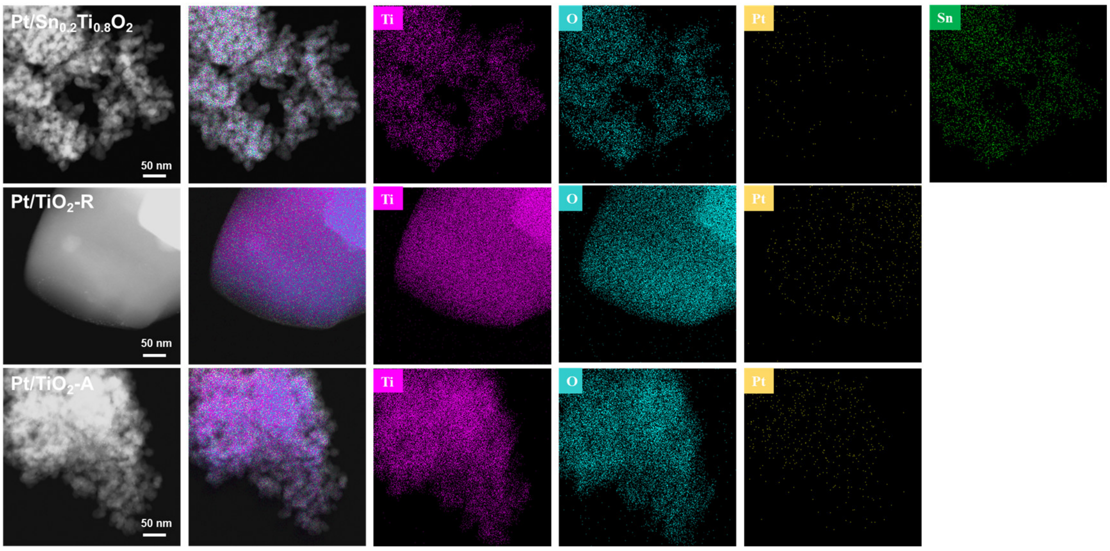

Supplementary Fig. S17 STEM-EDX elemental mapping images of Pt/$Sn_{0.2}Ti_{0.8}O_2$, Pt/TiO_2-R and Pt/TiO_2-A.

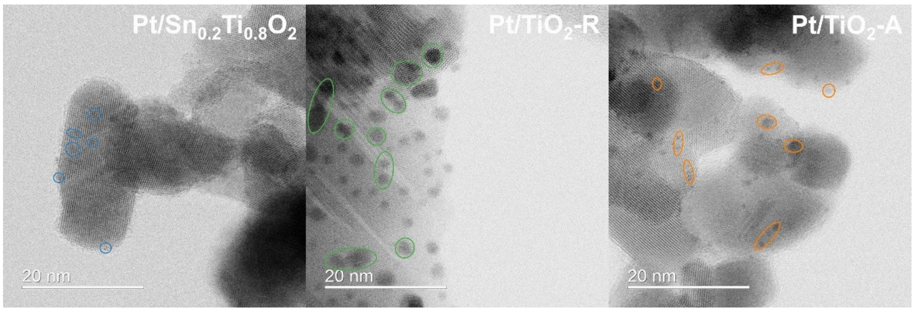

Supplementary Fig. S18 SAC-STEM HAABF images corresponding to those of Fig. 2d in the main text.

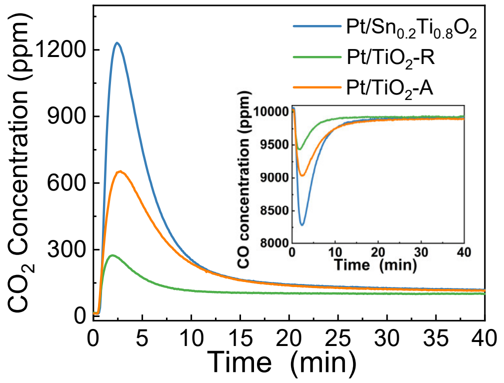

Supplementary Fig. S19 CO_2 generation and corresponding CO concentration (inset) as a function of time during the transient CO oxidation without O_2 supply (1% CO/N_2) at 200 °C.

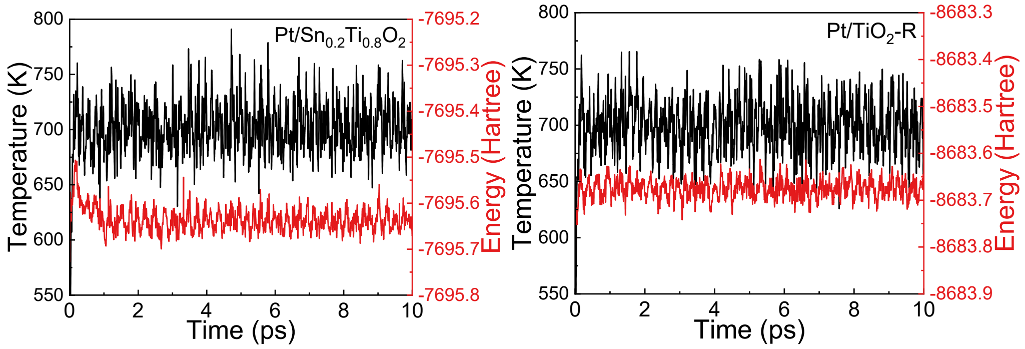

Supplementary Fig. S20 The simulation temperature and total energy during 10 ps of AIMD stability simulation over Pt/$Sn_{0.2}Ti_{0.8}O_2$ and Pt/TiO_2-R.

Supplementary Table S5 Total energy optimized by VASP using the initial configurations taken from 2–10 ps of AIMD simulation.

Sample	2 ps/eV	4 ps/eV	6 ps/eV	8 ps/eV	10 ps/eV
Pt/$Sn_{0.2}Ti_{0.8}O_2$	-1917.73	-1917.72	-1917.73	-1917.73	-1917.73
Pt/TiO_2-R	-2022.77	-2022.73	-2022.79	-2022.80	-2022.73

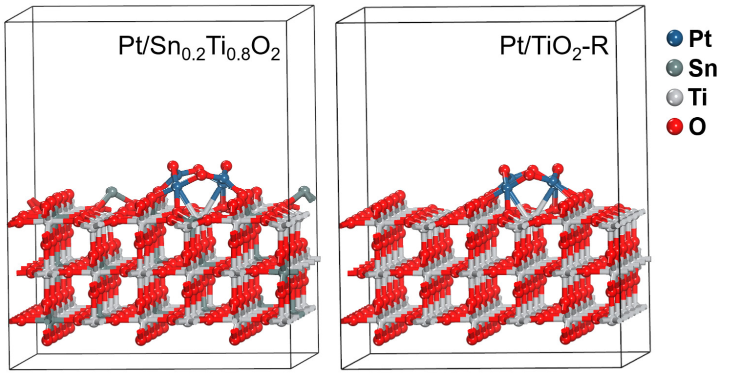

Supplementary Fig. S21 Complete configurations of Pt/$Sn_{0.2}Ti_{0.8}O_2$ and Pt/TiO_2-R optimized by VASP using the initial configuration taken from AIMD simulation at 8 ps.

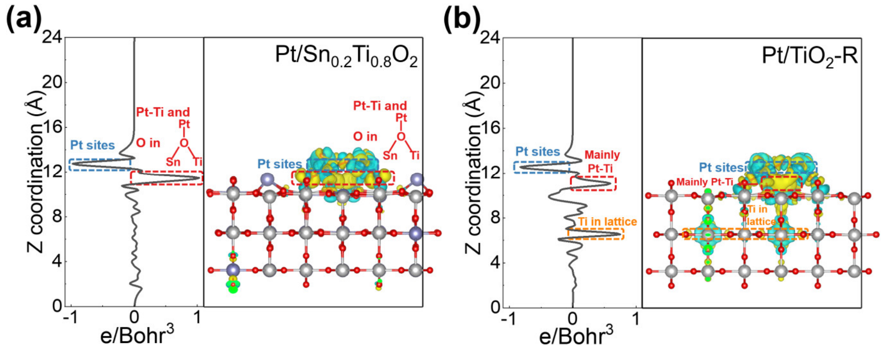

Supplementary Fig. S22 Difference of charge density and the corresponding planar-average charge density analysis of Pt_4O_4 clusters loaded on (a) $Sn_{0.2}Ti_{0.8}O_2$ and (b) TiO_2-R. The charge density difference was calculated by the equation $\Delta\rho = \rho_{AB}$-ρ_A-ρ_B, where A represents Pt_4O_4 clusters, and B represents $Sn_{0.2}Ti_{0.8}O_2$ or TiO_2-R. (Yellow represents increase of electron density, and blue represents decrease of electron density).

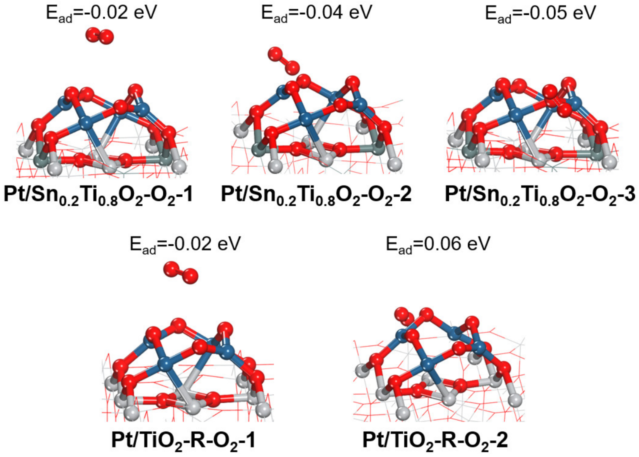

Supplementary Fig. S23 Adsorption energy of O_2 on Pt/$Sn_{0.2}Ti_{0.8}O_2$ and Pt/TiO_2-R.

Supplementary References

1. Somorjai, G. A. & Park, J. Y. Molecular factors of catalytic selectivity. *Angew. Chem. Int. Edit.* **47**, 9212-9228 (2008).
2. Ozturk, O. *et al.* Thermal decomposition of generation-4 polyamidoamine dendrimer films: Decomposition catalyzed by dendrimer-encapsulated Pt particles. *Langmuir* **21**, 3998-4006 (2005).
3. Ye, H., Scott, R. W. & Crooks, R. M. Synthesis, characterization, and surface immobilization of platinum and palladium nanoparticles encapsulated within amine-terminated poly(amidoamine) dendrimers. *Langmuir* **20**, 2915-2920 (2004).
