# Supplementary material for: Reverse oxygen spillover triggered by CO adsorption on Sn-doped Pt/TiO2 for low-temperature CO oxidation
Source: Nat Commun. 2023 Jun 13;14:3477. doi: 10.1038/s41467-023-39226-6 (PMC10264398; doi:10.1038/s41467-023-39226-6)
Supplement: Supplementary file 3 — Description of Additional Supplementary Files [file 41467_2023_39226_MOESM3_ESM.pdf]

Description of Additional Supplementary Files

File Name: Supplementary Movie 1
Description: AIMD simulation of reverse O spillover among Pt/$Sn_{0.2}Ti_{0.8}O_2$ triggered by CO adsorption at 700K.

File Name: Supplementary Movie 2
Description: AIMD simulation of Pt/TiO_2-R after CO adsorption at 700K.
